# Targeted Liposomes Encapsulating miR-603 Complexes Enhance Radiation Sensitivity of Patient-Derived Glioblastoma Stem-Like Cells

**DOI:** 10.3390/pharmaceutics13081115

**Published:** 2021-07-21

**Authors:** Ahmed M. Shabana, Beibei Xu, Zachary Schneiderman, Jun Ma, Clark C. Chen, Efrosini Kokkoli

**Affiliations:** 1 Institute for NanoBioTechnology, Johns Hopkins University, Baltimore, MD 21218, USA; ahmed.shabana@jhu.edu (A.M.S.); zschnei1@jhu.edu (Z.S.); 2 Department of Pharmaceutics and Industrial Pharmacy, Faculty of Pharmacy, Cairo University, Cairo 11562, Egypt; 3 Department of Neurosurgery, University of Minnesota Medical School, Minneapolis, MN 55455, USA; bxuphd@gmail.com (B.X.); jma603@umn.edu (J.M.); 4 Department of Chemical and Biomolecular Engineering, Johns Hopkins University, Baltimore, MD 21218, USA

**Keywords:** stealth liposomes, microRNA delivery, targeting integrin α_5_β_1_, miR-603, glioblastoma stem-cell state, radiation therapy

## Abstract

Despite potential for clinical efficacy, therapeutic delivery of microRNAs (miRNA) remains a major translational barrier. Here, we explore a strategy for miRNA delivery in the treatment of glioblastoma, the most common form of adult brain cancer, that involves complexation of miRNA with polyethylenimine (PEI) and encapsulation in targeted liposomes. miRNA 603 (miR-603) is a master regulatory miRNA that suppresses glioblastoma radiation resistance through down-regulation of insulin-like growth factor 1 (IGF1) signaling. miR-603 was complexed with PEI, a cationic polymer, and encapsulated into liposomes decorated with polyethylene glycol (PEG) and PR_b, a fibronectin-mimetic peptide that specifically targets the α_5_β_1_ integrin that is overexpressed in glioblastomas. Cultured patient-derived glioblastoma cells internalized PR_b-functionalized liposomes but not the non-targeted liposomes. The integrin targeting and complexation of the miRNA with PEI were associated with a 22-fold increase in intracellular miR-603 levels, and corresponding decreases in IGF1 and IGF1 receptor (IGF1R) mRNA expression. Moreover, treatment of glioblastoma cells with the PR_b liposomes encapsulating miR-603/PEI sensitized the cells to ionizing radiation (IR), a standard of care treatment for glioblastomas. These results suggest that PR_b-functionalized PEGylated liposomes encapsulating miR-603/PEI complexes hold promise as a therapeutic platform for glioblastomas.

## 1. Introduction

MicroRNAs (miRNAs) play key roles in modulating therapeutic resistance of glioblastomas, the most common form of brain cancer in adults, and one of the deadliest of human cancers [1]. By binding to the 3′ untranslated regions of target mRNAs, these short (19–24 base pairs), noncoding RNAs dampen gene expression [2]. Master-regulatory miRNAs coordinate gene expression regulated by distinct transcriptional units toward a single phenotype [3]. RNA extracted from glioblastomas of patients pre- and post-treatment with standard-of-care therapy (ionizing radiation (IR)/temozolomide), showed that miR-603, miR-181d, and miR-124_3-p, were lower in the post-treatment specimens, with miR-603 demonstrating the greatest decrease [4]. In response to IR, miR-603 is packaged into extracellular vesicles and released, resulting in decreased intracellular miR-603, which in turn, simultaneously de-represses insulin-like growth factor 1 (IGF1) and IGF1 receptor (IGF1R) expression to induce radiation resistance [4]. This increase, in turn, promotes cancer stem-cell state and acquired radiation resistance in glioblastomas [4]. Exogenous expression or transfection of miR-603 suppresses ionizing radiation resistance [4], and represents an attractive therapeutic strategy, especially in light of recent approval of the first small interfering RNA (siRNA)-based therapy [5]. However, clinical use of miRNA is limited by several factors, including lack of stability in biological fluids as they can be degraded by nucleases, poor ability to cross cell membranes, and the potential of off-target effects [6,7]. Thus, nanoparticles that can provide solutions to these barriers by protecting and targeting miRNAs to specific cells, are needed.

Our group previously demonstrated that therapeutic delivery of siRNA can be achieved by liposome encapsulation of complexes of siRNA with polyethylenimine (PEI), a cationic polymer [8]. The siRNA/PEI complexes are encapsulated in the aqueous core of the liposomes, thus protecting the siRNA from nucleases. When these liposomes are decorated with cancer targeting peptides, they are selectively internalized by cancer cells, thus delivering the siRNA/PEI complexes selectively in the cancer cells and protecting healthy cells from any toxicity associated with the siRNA and cationic PEI. We previously demonstrated that complexation of siRNA/PEI in the liposomes was advantageous [8], as PEI facilitates escape from endosomes and lysosomes into the cytoplasm, thus resulting in increased targeted gene suppression. miR-603 has been previously encapsulated in liposomes [9,10]. Given the functional and structural similarity between siRNA and miRNA, we propose the feasibility of the siRNA/PEI strategy in therapeutic miRNA delivery. In this context, we generated liposomes that encapsulated miR-603/PEI complexes in their aqueous core and decorated the liposomes with the PR_b peptide, a fibronectin-mimetic peptide that binds to integrin α_5_β_1_ with high affinity and specificity [11]. PR_b was selected because α_5_β_1_ is highly over-expressed in multidrug-resistant glioblastoma cells [12,13]. We also chose to work with patient-derived glioblastoma stem-like cells, as serum cultured, adherent glioblastoma lines poorly recapitulate cancer heterogeneity and therapeutic resistance [14,15].

Here, we demonstrate that patient-derived glioblastoma cell lines, grown under conditions that induce stem-cell like properties [16], selectively internalize PR_b-functionalized liposomes encapsulating miR-603/PEI complexes. This uptake is associated with a 22-fold increase in miR-603 that suppressed expression of IGF1 and IGF1R mRNA levels. Phenotypically, treatment of patient-derived glioblastoma stem-like cells with PR_b liposomes encapsulating miR-603/PEI complexes induced significant radiation sensitivity compared to non-targeted liposomes and liposomes that encapsulated free miR-603. To our knowledge, this study is the first to demonstrate proof-of-principle for the therapeutic delivery of miRNA using PEI complexation and targeted liposomes as a platform to augment radiation sensitivity of glioblastoma cells.

## 2. Materials and Methods

### 2.1. Materials and Cells

Fluorescently labeled miR-603 was purchased from Horizon Discovery Biosciences LTD (Boulder, CO, USA), PR_b peptide was purchased from Biomatik (Wilmington, DE, USA), branched polyethyleneimine (PEI) 25 kDa, sephadex G-50 and calcein were purchased from Sigma-Aldrich (Burlington, MA, USA). Dipalmitoylphosphatidylcholine (DPPC), cholesterol and 1,2-dipalmitoyl-sn-glycero-3-phosphoethanolamine-*N*-[methoxy(polyethylene glycol)-750] (DPPE-PEG750) (ammonium salt) were purchased from Avanti Polar Lipids (Alabaster, AL, USA). Dialysis membrane 1000 kDa MWCO was purchased from Thermo Fisher Scientific (Waltham, MA, USA). Glioblastoma patient-derived cells grown under conditions that induce stem-cell like properties [16], GBM-CCC-001, GBM-CCC-002, GBM-CCC-003, were provided by Dr. Clark Chen (University of Minnesota, Minneapolis, MN, USA). The cells were passaged as neurospheres in Dulbecco’s modified Eagle’s medium/F-12 supplemented with 20 ng/mL recombinant human (rh) epidermal growth factor (EGF), 10 ng/mL rh basic fibroblast growth factor (bFGF), 2 µg/mL heparin, B-27 supplement, 100 U/mL penicillin and 100 ng/mL streptomycin in ultra-low attachment flasks in 37 °C incubator with 5% CO_2_. Supplies for cell culture were purchased from Thermo Fisher Scientific (Waltham, MA, USA). A representative image of a neurosphere is shown in Figure S1.

### 2.2. Preparation and Characterization of miR-603/PEI Complexes

Fluorescently labeled miR-603 (active strand: 5′-P-CACACACUGCAAUUACUUUU GC-3′, passenger strand: 5′-fluorescein-AAAAGUAAUUGCAGUGUGUGUU-3′) was complexed using branched PEI 25 kDa. Then, 200 nM miR-603 dissolved in 6 mM HEPES buffer (pH 7.4) was mixed with an equal volume of PEI in 6 mM HEPES buffer (pH 7.4) at different nitrogen to phosphate (N:P) ratios to yield a final miR-603 concentration of 100 nM. The mixture was vortexed for 5 s followed by 30 min incubation period at room temperature. The size and zeta potential of the miR-603/PEI complexes were measured using a Zetasizer (Malvern Panalytical, Westborough, MA, USA).

### 2.3. Preparation and Characterization of Liposomes

Liposomes were prepared using the dry lipid film technique [8]. Stock solutions of DPPC, cholesterol and DPPE-PEG750 in chloroform were mixed in the following molar ratio 64:35:1 respectively, to give a total lipid concentration of 10 mM. The PR_b peptide (KSSPHSRNSGSGSGSGSGRGDSP) was purchased from Biomatik (Wilmington, DE, USA) and the C_16_ double tail peptide-amphiphile was synthesized and characterized by electrospray ionization mass spectrometry (ESI-MS) as previously described [17]. To prepare integrin targeted liposomes, the PR_b-amphiphile was mixed with the other liposomal components at an initial concentration of 5 mol% (DPPC:cholesterol:DPPE-PEG750:PR_b-amphiphile at 59:35:1:5 mol%). The lipid mixture was placed in a rotary evaporator flask at 50 °C to remove the organic solvent, followed by further drying the sample under vacuum overnight to generate a uniform dry lipid film. To prepare calcein-loaded liposomes, the lipid film was hydrated with 2 mM calcein at 45 °C for 2 h. The generated multilamellar vesicles were subjected to extrusion through 200 nm polycarbonate membrane for 21 cycles at 45 °C. The unencapsulated dye was removed by gel permeation chromatography using a Sephadex G-50 packed column. To prepare miR-603 or miR-603/PEI-loaded liposomes, the lipid film was hydrated with 1 mL of either 100 nM miR-603 or miR-603/PEI complex solution in 6 mM HEPES buffer pH 7.4 at 45 °C for 2 h. The generated multilamellar vesicles were extruded as mentioned above and the unencapsulated miR-603 or miR-603/PEI complexes were removed through overnight dialysis at 4 °C using a 1000 kDa MWCO dialysis membrane. The size and zeta potential of all generated liposomes were measured using a Zetasizer (Malvern Panalytical, Westborough, MA, USA). The final peptide concentration on the surface of the liposomes was evaluated using a BCA protein assay (Thermo Fisher Scientific, Waltham, MA, USA) following the manufacturer’s protocol. The encapsulation efficiency of miR-603 was determined by fluorescence spectroscopy using a microplate reader (BioTek Synergy H1, Winooski, VT, USA).

### 2.4. Cryogenic Transmission Electron Microscopy (Cryo-TEM)

The morphology of miR-603/PEI complexes at N:P ratio of 6:1 and miR-603/PEI-loaded liposomes was examined with cryo-TEM. Briefly, 5 µL of an aqueous suspension of either miR-603/PEI or liposomes encapsulating the complexes were deposited onto carbon copper grids (Ted Pella, Redding, CA, USA) pretreated with glow discharge for 40 s and vitrified in liquid ethane by Vitroblot using the following parameters (4 s blot time, 0 offset, 0 s wait time, 0 s drain time, 100% relative humidity). The prepared sample grids were stored under liquid nitrogen until they were transferred to a FEI Tecnai-12 TEM operated with an acceleration voltage of 100 keV (Integrated Imaging Center, Institute for NanoBioTechnology, Johns Hopkins University, Baltimore, MD, USA). Images were acquired using an Eagle 2k CCD camera and SIS Megaview III wide-angle CCD camera.

### 2.5. Expression of Integrin α_5_β_1_

The cell expression of integrin α_5_β_1_ in glioblastoma neurospheres was verified as previously described [18]. GBM-CCC-001, GBM-CCC-002 and GBM-CCC-003 neurospheres were collected into 15 mL Falcon tubes, pelleted down, and dissociated into single cells. After cell counting, 200,000 cells were transferred into Eppendorf tubes and incubated with the primary anti-integrin α_5_β_1_ antibody MAB1969 (Sigma-Aldrich, Burlington, MA, USA) or mouse IgG isotype control (Sigma-Aldrich, Burlington, MA, USA) at 1:100 dilution in PBSA (1% *w/v* bovine serum albumin (BSA) in phosphate buffer saline (PBS)) at 4 °C for 30 min. Following the incubation period, cells were pelleted and washed twice with ice-cold PBSA, then incubated with the FITC-conjugated anti-mouse IgG secondary antibody (Thermo Fisher Scientific, Waltham, MA, USA) for 30 min at 4 °C Finally, cells were pelleted and washed twice with ice-cold PBSA, and flow cytometric analysis was performed immediately using BD FACSCanto (Integrated Imaging Center, Institute for NanoBioTechnology, Johns Hopkins University, Baltimore, MD, USA). Cell autofluorescence was subtracted from all measurements.

### 2.6. Flow Cytometry

Glioblastoma neurospheres (GBM-CCC-001, GBM-CCC-002 and GBM-CCC-003) were collected into 15 mL Falcon tubes, pelleted down, and dissociated into single cells. After cell counting, 200,000 cells were transferred into Eppendorf tubes in neurosphere culture media and treated with 2 mM calcein-loaded PR_b liposomes or non-targeted liposomes at a lipid concentration of 150 µM for 48 h at 37 °C on a shaker. After the incubation period, cells were pelleted down and washed twice with PBS. Flow cytometric analysis was carried out immediately using BD FACSCanto. Non-treated cells that received only media served as a control. Cell autofluorescence was subtracted from all measurements. For the blocking experiment, the same procedure was followed, except cells were preincubated with 1 mg/mL free PR_b peptide in media for 1 h at 37 °C prior to addition of the PR_b liposomes and incubation for an additional 1 h at 37 °C.

### 2.7. Confocal Microscopy

Glioblastoma neurospheres (GBM-CCC-001, GBM-CCC-002 and GBM-CCC-003) were collected into 15 mL Falcon tubes, pelleted down, and dissociated into single cells. After cell counting, 200,000 cells were transferred into Eppendorf tubes in neurosphere culture media and treated with 2 mM calcein-loaded PR_b liposomes or non-targeted liposomes at a lipid concentration of 150 µM for 48 h at 37 °C on a shaker. Cells were then pelleted down, washed twice with PBS and mounted onto coverslips in a 12 well plate via centrifugation at 300 g for 10 min. Cells were fixed using 4% paraformaldehyde solution in PBS for 30 min. Nuclear staining was carried out using the cell membrane permeable dye Hoechst 33342 (Thermo Fisher Scientific, Waltham, MA, USA) at a concentration of 2.0 µg/mL, and the cell membrane was stained with cell impermeable AlexaFluor647 wheat germ agglutinin (Thermo Fisher Scientific, Waltham, MA, USA) at 5.0 µg/mL in PBS for 15 min. Cells were mounted onto glass slides using Prolong Gold and imaged with a Carl Zeiss LSM700 confocal microscope (Integrated Imaging, Institute for NanoBioTechnology, Johns Hopkins University, Baltimore, MD, USA).

### 2.8. RNA Extraction and Quantitative Real-Time Polymerase Chain Reaction (qRT-PCR)

GBM-CCC-001 neurospheres were pelleted down and dissociated into single cells. Then, 200,000 cells were seeded in Eppendorf tubes and incubated with either PBS (control), PR_b empty liposomes, or PR_b liposomes encapsulating miR-603 or miR-603/PEI complexes (final working concentration of miR-603 was 100 nM) for 48 h on a shaker at 37 °C. Total RNA was isolated from cells using miRNeasy mini kit (217004, Qiagen, Germantown, MD, USA). cDNA synthesis was performed using the miScript II RT kit (218160, Qiagen, Germantown, MD, USA) according to the manufacturer’s protocol. mRNA transcripts were quantified using SYBR Green (Bio-Rad, Hercules, CA, USA) on the Bio-Rad CFX96 Real-Time PCR Detection System. The following qPCR primers were used:

Hs_miR-603_3 miScript Primer (MS00037933, Qiagen, Germantown, MD, USA),
IGF1: 5′-GCAGCACTCATCCACGATGC-3′ (forward primer),
5′-TGTGGAGACAGGGGCTTTTATTTC-3′ (reverse primer).
IGF1R: 5′-AAGTTCTGGTTGTCGAGGA-3′ (forward primer),
5′-GAGCAGCTAGAAGGGAATTAC-3′ (reverse primer).
18s: 5′-TTGCCCTCCAATGGATCCT-3′ (forward primer),
5′-GGGAGGTAGTGACGAAAAATAACAAT-3′ (reverse primer).

### 2.9. Limiting Dilution Assay

GBM-CCC-001 neurospheres were pelleted down and dissociated into single cells. Then, 200,000 cells were seeded in Eppendorf tubes and incubated with either PBS (control), PR_b empty liposomes, or PR_b liposomes encapsulating miR-603 or miR-603/PEI complexes (final working concentration of miR-603 was 100 nM) for 24 or 48 h on a shaker at 37 °C. After incubation, cells were irradiated with X-rays (6 Gy) and briefly washed with PBS. The setting of the X-ray irradiator (X-RAD 320, Precision X-Ray, North Branford, CT, USA) was: set dose 6 Gy, 22 °C, 101.3 kPa, 320 KV, 12.5 mA. Once the set dose was reached, IR stopped automatically. Single-cell suspensions of GBM-CCC-001 cells were then prepared, serially diluted, and plated into 96-well ultra-low attachment plates containing 100 µL neurosphere culture media at various seeding densities. After two weeks, each well was scored for the absence or presence of tumor spheres by visual inspection (at least one aggregate of ≥50 cells). Data were analyzed using Extreme Limiting Dilution Analysis (ELDA) [19].

## 3. Results and Discussion

### 3.1. Encapsulation of miR-603/PEI Complexes into PR_b Liposomes

Anionic miR-603 was complexed with the cationic branched PEI at different nitrogen to phosphate (N:P) ratios. Dynamic light scattering (DLS) was used to measure the hydrodynamic diameter of the complexes, and zeta potential was evaluated via electrophoretic light scattering. The results are shown in Table S1. The zeta potential increased from −19 to 20 mV on average as the N:P ratio increased. Our previous study demonstrated that siRNA/PEI complexes at N:P ratio of 6 provided the most efficient mRNA silencing [8]. DLS showed only one peak for N:P = 6 complexes, with the smallest polydispersity index (PDI) of 0.11 ± 0.04. Their hydrodynamic diameter was 103.9 ± 24.4 nm, and the zeta potential was 20.2 ± 1.1 mV. As such, complexes at N:P = 6 were used.

Targeted and non-targeted liposomes were prepared encapsulating miR-603 or miR-603/PEI complexes. These particles were characterized in terms of size, zeta potential and encapsulation efficiency (Table 1). The nanoparticles are intended for local delivery to glioblastoma and all liposomes used in this study had 1 mol% DPPE-PEG750 to prevent aggregation of the nanoparticles. The peptide concentration on the surface of the PR_b-functionalized liposomes was 3.1 ± 0.5 mol%. The size of the liposomes was similar for all formulations tested. The presence of the positively charged PR_b peptide on the surface of the nanoparticles had an effect on the zeta potential of the liposomes, as it went from −25 mV for non-targeted formulations to about 5 mV for the PR_b-functionalized liposomes. The encapsulation efficiency of miR-603 was about 70% for both targeted and non-targeted formulations. This efficiency decreased to 44% and 58% when miR-603 was encapsulated as complexes with PEI in non-targeted and targeted liposomes, respectively.

**Figure 1:**
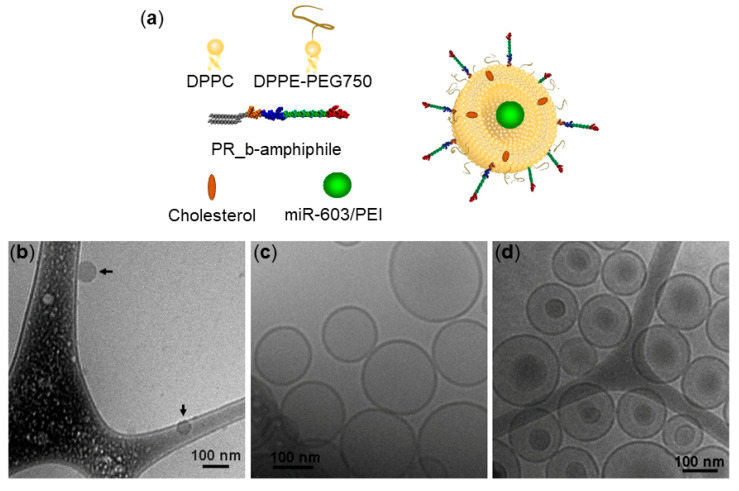
(**a**) Components and schematic of the PR_b-functionalized liposome encapsulating a miR-603/PEI complex. Not drawn to scale. Cryo-TEM images of (**b**) miR-603/PEI complexes at N:P = 6; (**c**) empty PR_b liposomes; (**d**) PR_b liposomes encapsulating miR-603/PEI complexes at N:P = 6.

PR_b liposomes encapsulating miR-603/PEI complexes at N:P = 6 were visualized via cryo-TEM (Figure 1). For comparison, cryo-TEM of empty PR_b liposomes and free complexes are also shown. Cryo-TEM images verified the presence of encapsulated miR-603/PEI complexes in unilamellar liposomes. The complexes had a diameter of 68 ± 14 nm, the empty PR_b liposomes were 136 ± 34 nm, and the diameter of the liposomes with the encapsulated complexes was 141 ± 34. There was no significant statistical difference between the diameters of the empty liposomes and the liposomes with the complexes. Differences between DLS and cryo-TEM measurements can be attributed to the fact that DLS average diameters are derived from a Cumulants analysis of the measured intensity correlation curve, wherein a single particle size is assumed and only the initial part of the correlation function is force-fitted to a single exponential decay. In addition, DLS results measure the average size by scattering intensity, whereas the cryo-TEM size is the average by number. Thus, average sizes obtained from DLS can be slightly larger than measurements obtained from cryo-TEM images.

### 3.2. Targeting Patient-Derived Glioblastoma Cell Lines with PR_b Liposomes

PR_b is a fibronectin-mimetic peptide that exhibits highly selective binding to the α_5_β_1_ integrin [11,20]. α_5_β_1_ integrin is a promising target that has been shown to be overexpressed on cancer cells as well as on cancer vasculature, and minimally expressed on normal healthy tissues [21,22,23,24]. It is associated with increased tumorigenicity, malignancy and tumor cell invasiveness [21,25,26]. In addition, α_5_β_1_ is also expressed on certain primary cell lines, but its expression is downregulated during development [27]. PR_b-functionalized liposomes internalize into cancer cells primarily through caveolae-mediated endocytosis [17], in agreement with other reports showing that caveolin-1 constitutively regulates endocytosis of α_5_β_1_ [28,29].

**Figure 2:**
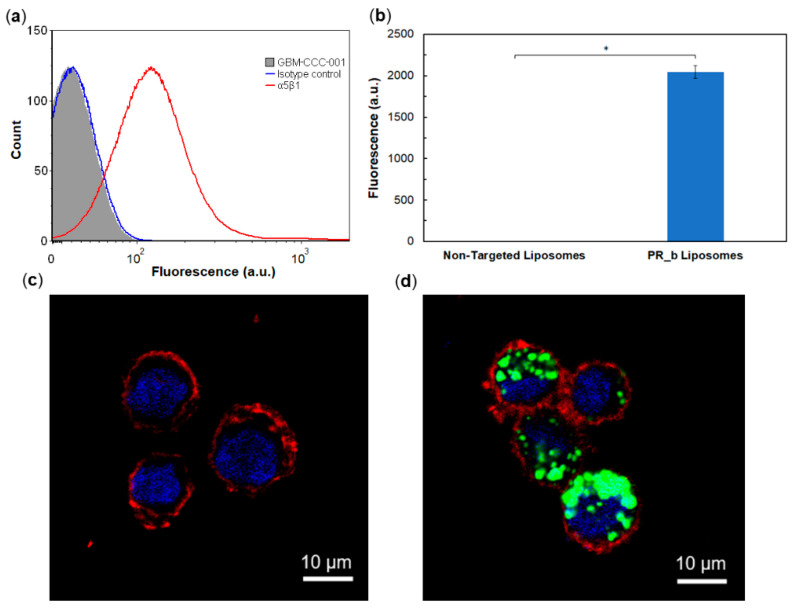
(**a**) Representative integrin α_5_β_1_ expression in GBM-CCC-001 cells. (**b**) Cell association of non-targeted liposomes and PR_b-functionalized liposomes was evaluated via flow cytometry in GBM-CCC-001 cells after 48 h incubation at 37 °C. Data are presented as mean ± SD (*n* = 2). Student’s t-test analysis was used to determine significance, * *p* < 0.05. Confocal microscopy images of (**c**) non-targeted liposomes and (**d**) PR_b-functionalized liposomes incubated for 48 h at 37 °C with GBM-CCC-001 cells. Liposomes with encapsulated calcein are shown in green, cell membranes in red and nuclei in blue.

α_5_β_1_ is highly expressed on glioblastomas [12,13]. We confirmed the expression of the α_5_β_1_ integrin in three independent patient-derived glioblastoma lines, GBM-CCC-001, GBM-CCC-002 and GBM-CCC-003, cultured in conditions that induce cancer-stem cell phenotypes [16] (Figure S2). Figure 2a shows a representative flow cytometry histogram of GBM-CCC-001 cells. The PR_b peptide-amphiphile was synthesized by conjugating the PR_b peptide to a C_16_ dialkyl tail as previously described [11], and was used in the preparation of the PR_b-functionalized liposomes. The association of PR_b liposomes and non-targeted liposomes with the glioblastoma stem-like cells was investigated by flow cytometry (Figure 2b and Figure S3). The fluorescence signal from the non-targeted liposomes was negligible for the glioblastoma lines tested, while a notable increase in fluorescence signal was observed in the cells treated with PR_b liposomes. A blocking experiment with free PR_b peptide (Figure S4) showed significant reduction in PR_b liposome binding to glioblastoma cells, demonstrating specificity of the PR_b peptide for the α_5_β_1_ integrin [20].

Cell internalization of the targeted and non-targeted liposomes was further investigated with confocal microscopy. Figure 2c and Figure S5 show no detectable uptake of non-targeted liposomes by the patient-derived glioblastoma lines. In contrast, all lines tested showed internalization of the PR_b liposomes. Together, these results support the key role of the PR_b peptide in facilitating the binding and internalization of PEGylated liposomes in patient-derived glioblastoma lines.

### 3.3. PR_b Liposomes Encapsulating miR-603/PEI Complexes Suppress the Expression of IGF1 and IGF1R in Patient-Derived Glioblastoma Cells

**Figure 3:**
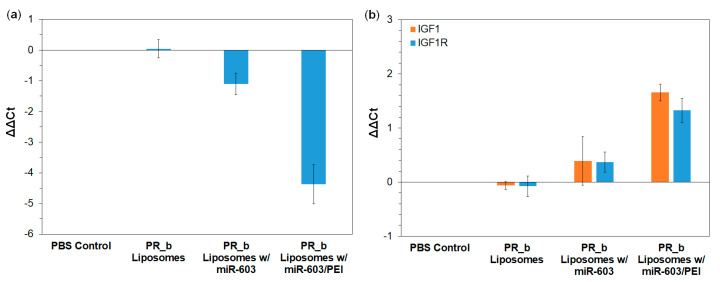
mRNA levels of (**a**) miR-603 and (**b**) IGF1 and IGF1R relative to PBS control determined by qRT-PCR in GBM-CCC-001 cells following exposure to indicated treatments for 48 h at 37 °C. Data are presented as mean ± SD (*n* = 3). *p*-values from one-way ANOVA with Tukey’s HSD post-hoc analysis can be found in Tables S2–S4.

IGF1 and IGF1R are down-stream targets of miR-603 [4]. Therefore, we tested whether uptake of PR_b liposomes encapsulating miR-603/PEI complexes affected the expressions of miR-603, IGF1 and IGF1R in the GBM-CCC-001 cells. After 48 h of treatment, PBS control and empty PR_b liposomes had no effect on cellular levels of miR-603, IGF1 or IGF1R (Figure 3). PR_b liposomes ecapsulating free miR-603 showed a 2.2-fold increase in cellular miR-603 level (Figure 3a). The fold difference (1.3-fold decrease) in the IGF1 and IGF1R mRNA expression was not significant compared to the PBS control (Figure 3b). In contrast, PR_b liposomes ecapsulating miR-603/PEI complexes induced a 22.1-fold increase in cellular miR-603 (Figure 3a). This increase was associated with a 3.2- and 2.5-fold decrease in IGF1 and IGF1R mRNA expression, respectively (Figure 3b). These results are generally consistent with previous reports suggesting that PEI facilitates escape of cargo from endosomes and lysosomes [8]. PEI is believed to facilitate endosomal escape through a proton sponge effect, however, the actual mechanism associated with cargo escape from endosomes is the subject of debate [30,31,32]. Taken together, our data support the PEI-liposome platform as a potential therapeutic delivery strategy.

### 3.4. PR_b Liposomes Encapsulating miR-603/PEI Complexes Enhance Radiation Sensitivity of Patient-Derived Glioblastoma Cells

IGF1 and IGF1R signaling play an essential role in augmenting glioblastoma resistance to IR, a standard of care treatment for glioblastomas. Given that PR_b liposomes encapsulating miR-603/PEI complexes suppressed the expression of IGF1 and IGF1R, we next tested whether such treatment enhanced the IR sensitivity of patient-derived glioblastoma cells. GBM-CCC-001 were treated with PR_b liposomes encapsulating free miR-603 or miR-603/PEI complexes for 24 and 48 h. The viabilities of these cells were assessed using a standard limiting dilution assay [4]. In this assay, single cells were plated into 96-well plates after mock or IR treatment at various seeding densities and allowed to grow for two weeks. Higher seeding density required for sphere formation, or a rightward shift in the curve, implies decreased proliferative potential.

**Figure 4:**
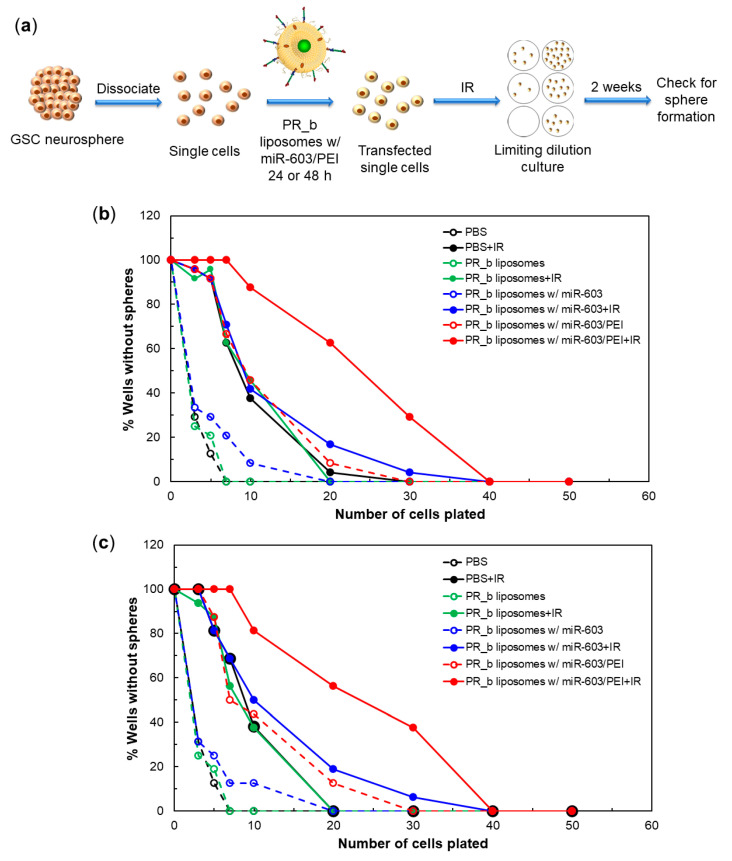
(**a**) Schematic representation of the limiting dilution assay. Limiting dilution analysis measured the clonogenic potential of GBM-CCC-001 cells, which were exposed to indicated liposomal formulations for either (**b**) 24 h or (**c**) 48 h with or without additional IR treatment. Limiting dilution assays were performed with 12 replicate wells (**b**) or 8 replicate wells (**c**) per dilution. The entire experiment was independently repeated twice. Statistical analysis was performed using the ELDA software and *p*-values can be found in Tables S5 and S6.

The treatment scheme of the radiation sensitivity experiment is summarized in Figure 4a. Expectedly, treatment with 6 Gy of IR resulted in an increase in the seeding density required for sphere formation, associated with a rightward shift in the proliferative potential curve. No significant change in seeding density requirement was observed if the radiation was carried out 24 or 48 h after treatment with PBS, empty liposomes, or PR_b liposomes encapsulating free miR-603 (Figure 4b,c). Results suggest these treatments did not significantly influence glioblastoma growth potential or radiation sensitivity. In contrast, treatment with PR_b liposomes encapsulating miR-603/PEI complexes for 24 or 48 h significantly increased the seeding density required for sphere formation (shifted the sphere formation curve to the right), suggesting that the treatment enhanced radiation sensitivity (Figure 4b,c).

Quantitative analysis of the limiting dilution assay using the ELDA software is shown in Table 2 [19]. This analysis indicates that treatment with PR_b liposomes encapsulating miR-603/PEI induced a 3-fold increase in radiation sensitivity relative to treatment with PBS, empty PR_b liposomes, or PR_b liposomes encapsulating free miR-603. Similar results were observed after 24 or 48 h incubation. Of note, without radiation, treatment with PR_b liposomes encapsulating miR-603/PEI increased the seeding density required for sphere formation 3 to 5-fold relative to PBS, empty PR_b liposomes, or PR_b liposomes with free miR-603 for both time points. This finding is consistent with our previous results that miR-603 suppressed the glioblastoma stem cell state and the associated proliferative potential in the absence of radiation [4].

## 4. Conclusions

We conducted a proof-of-principle study demonstrating the utility of targeted liposomes encapsulating miRNA/PEI complexes as a strategy for therapeutic delivery of miR-603. PR_b-functionalized liposomes encapsulating miR-603/PEI complexes afford intracellular delivery of miR-603, resulting in down-regulation of IGF1 and IGF1R, and subsequent radiation sensitization. Intra-tumoral injection of such liposomes has the potential to enhance the efficacy of IR, a standard-of-care treatment for glioblastoma, and yield meaningful improvement in clinical outcome.

## Figures and Tables

**Table 1:** Characterization of liposomes encapsulating miR-603 or miR-603/PEI complexes at N:P = 6.

Formulation	Encapsulating Load	Diameter (nm) ^1^	PDI ^2^	Zeta Potential (mV)	Encapsulation Efficiency (%)
Non-Targeted Liposomes	miR-603	174.5 ± 2.0	0.025 ± 0.006	−25.8 ± 0.5	70 ± 4
Non-Targeted Liposomes	miR-603/PEI	173.6 ± 0.7	0.068 ± 0.008	−24.7 ± 2.2	44 ± 5
PR_b Liposomes	miR-603	176.8 ± 1.5	0.029 ± 0.002	6.3 ± 0.1	69 ± 7
PR_b Liposomes	miR-603/PEI	173.2 ± 3.8	0.036 ± 0.030	4.0 ± 1.3	58 ± 3

^1^ Hydrodynamic diameters are reported based on intensity from DLS. ^2^ Polydispersity index. Data are reported as mean ± SD; *n* = 3.

**Table 2:** Stem cell frequency after incubation with various liposomal formulations.

Treatment	24 h Liposomal Incubation	95% CI ^1^ for 24 h Incubation	48 h Liposomal Incubation	95% CI ^1^ for 48 h Incubation
PBS Control	0.38	(0.30, 0.47)	0.37	(0.28, 0.48)
PBS Control + IR	0.09	(0.07, 0.10)	0.09	(0.07, 0.11)
PR_b Liposomes	0.35	(0.28, 0.44)	0.36	(0.27, 0.47)
PR_b Liposomes + IR	0.08	(0.07, 0.10)	0.09	(0.07, 0.12)
PR_b Liposomes w/miR-603	0.23	(0.19, 0.29)	0.25	(0.19, 0.32)
PR_b Liposomes w/miR-603 + IR	0.07	(0.06, 0.09)	0.07	(0.05, 0.09)
PR_b liposomes w/miR-603/PEI	0.08	(0.06, 0.10)	0.08	(0.06, 0.11)
PR_b liposomes w/miR-603/PEI + IR	0.03	(0.03, 0.04)	0.03	(0.03, 0.05)

^1^ Confidence intervals are reported as (lower, upper) value. Data are calculated with the ELDA software from measurements shown in Figure 4b,c.

## Data Availability

Not applicable.

## Supplementary Materials

The following are available online at https://www.mdpi.com/article/10.3390/pharmaceutics13081115/s1, Figure S1: Representative bright field microscopy image of a GBM-CCC-001 neurosphere. Figure S2: α_5_β_1_ integrin expression in patient-derived glioblastoma stem-like cells. Figure S3: Cell association of non-targeted liposomes or PR_b-functionalized liposomes with GBM-CCC-002 and GBM-CCC-003 cells measured via flow cytometry. Figure S4: Blocking experiment. Cell association of PR_b-functionalized liposomes was measured via flow cytometry in GBM-CCC-001 cells pretreated with or without free PR_b peptide. Figure S5: Confocal microscopy images of cells incubated with liposomes for 48 h at 37 °C. Top row shows GBM-CCC-002 cells incubated with (**a**) non-targeted liposomes and (**b**) PR_b-functionalized liposomes. Bottom row shows GBM-CCC-003 cells incubated with (**c**) non-targeted liposomes and (**d**) PR_b-functionalized liposomes. Table S1: Characterization of miR-603/PEI complexes at different N:P ratios. Table S2: Statistical analysis comparing relative miR-603 mRNA level in GBM-CCC-001 cells following indicated treatments. Table S3: Statistical analysis comparing relative IGF1 mRNA level in GBM-CCC-001 cells following indicated treatments. Table S4: Statistical analysis comparing relative IGF1R mRNA level in GBM-CCC-001 cells following indicated treatments. Table S5: Statistical analysis comparing differences in stem cell frequency after 24 h incubation with liposomal formulations. Table S6: Statistical analysis comparing differences in stem cell frequency after 48 h incubation with liposomal formulations.
