# Supplementary material for: Targeted Liposomes Encapsulating miR-603 Complexes Enhance Radiation Sensitivity of Patient-Derived Glioblastoma Stem-Like Cells
Source: Pharmaceutics. 2021 Jul 21;13(8):1115. doi: 10.3390/pharmaceutics13081115 (PMC8399469; doi:10.3390/pharmaceutics13081115)
Supplement: Supplementary file 1 [file pharmaceutics-13-01115-s001.zip › pharmaceutics-1251894-supp-final.pdf]

pharmaceutics

Supplementary Materials: Targeted Liposomes Encapsulating miR-603 Complexes Enhance Radiation Sensitivity of Patient-Derived Glioblastoma Stem-Like Cells

Ahmed M. Shabana, Beibei Xu, Zachary Schneiderman, Jun Ma, Clark C. Chen and Efrosini Kokkoli

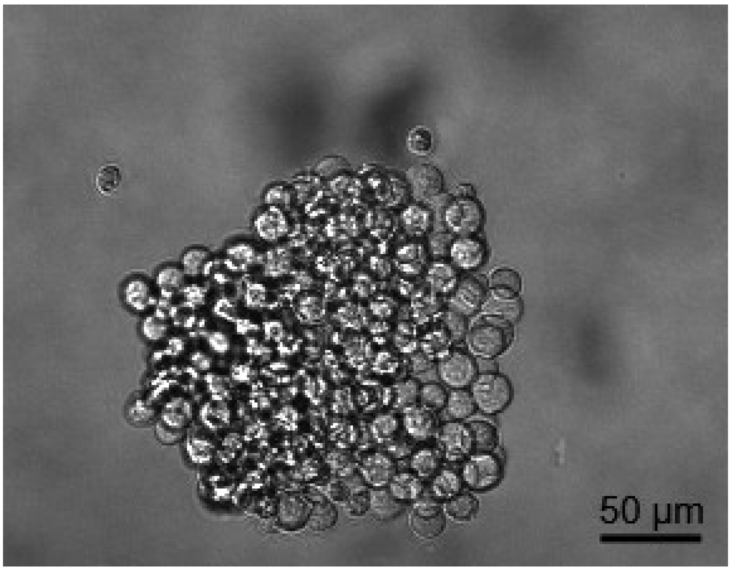

Figure S1. Representative bright field microscopy image of a GBM-CCC-001 neurosphere.

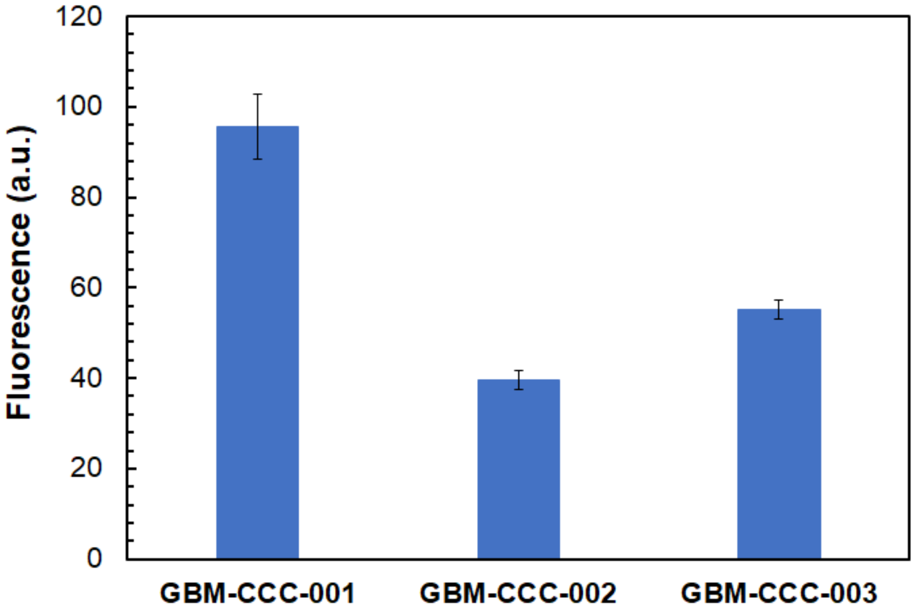

Figure S2. $\alpha_5\beta_1$ integrin expression in patient-derived glioblastoma stem-like cells. Data are presented as mean ± SD (n = 3).

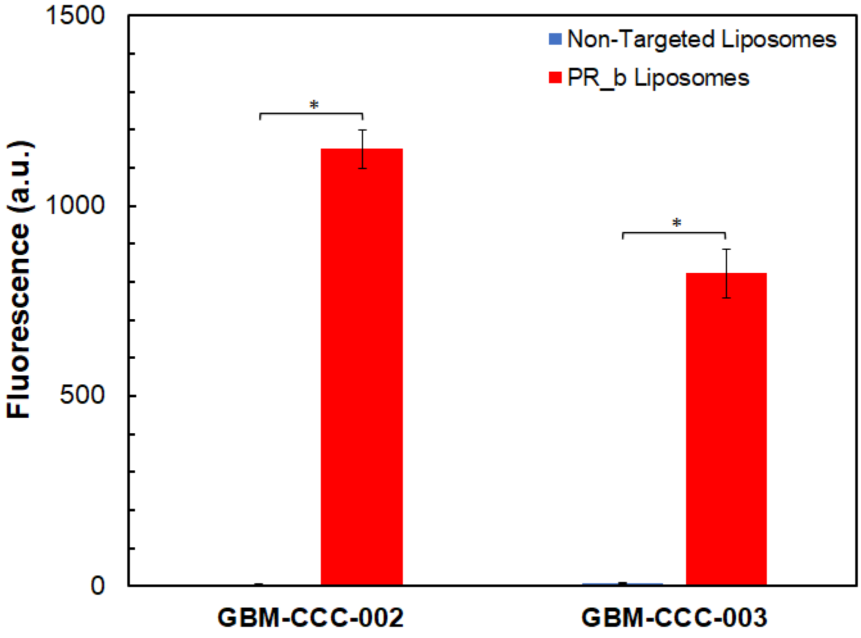

Figure S3. Cell association of non-targeted liposomes or PR_b-functionalized liposomes with GBM-CCC-002 and GBM-CCC-003 cells measured via flow cytometry. Cells were incubated with liposomes for 48 h at 37 °C. Data are presented as mean ± SD (n = 3). Student's t-test analysis was used to determine significance, * $p < 0.05$.

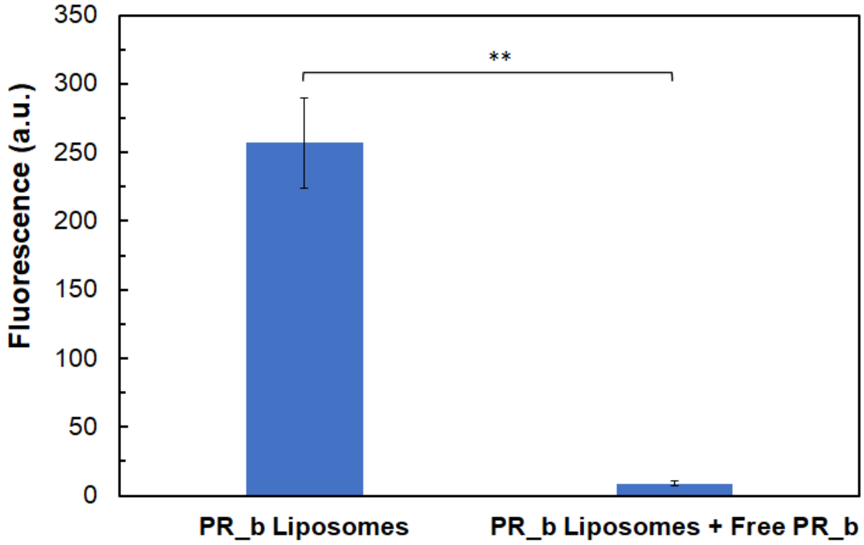

Figure S4. Blocking experiment. Cell association of PR_b-functionalized liposomes was measured via flow cytometry in GBM-CCC-001 cells pretreated with or without free PR_b peptide. Cell surface integrins were blocked by free PR_b peptide for 1 h at 37 °C before incubating the cells with PR_b liposomes for an additional 1 h at 37 °C. Data are presented as mean ± SD (n = 3). Student's t-test analysis was used to determine significance, ** $p < 0.01$.

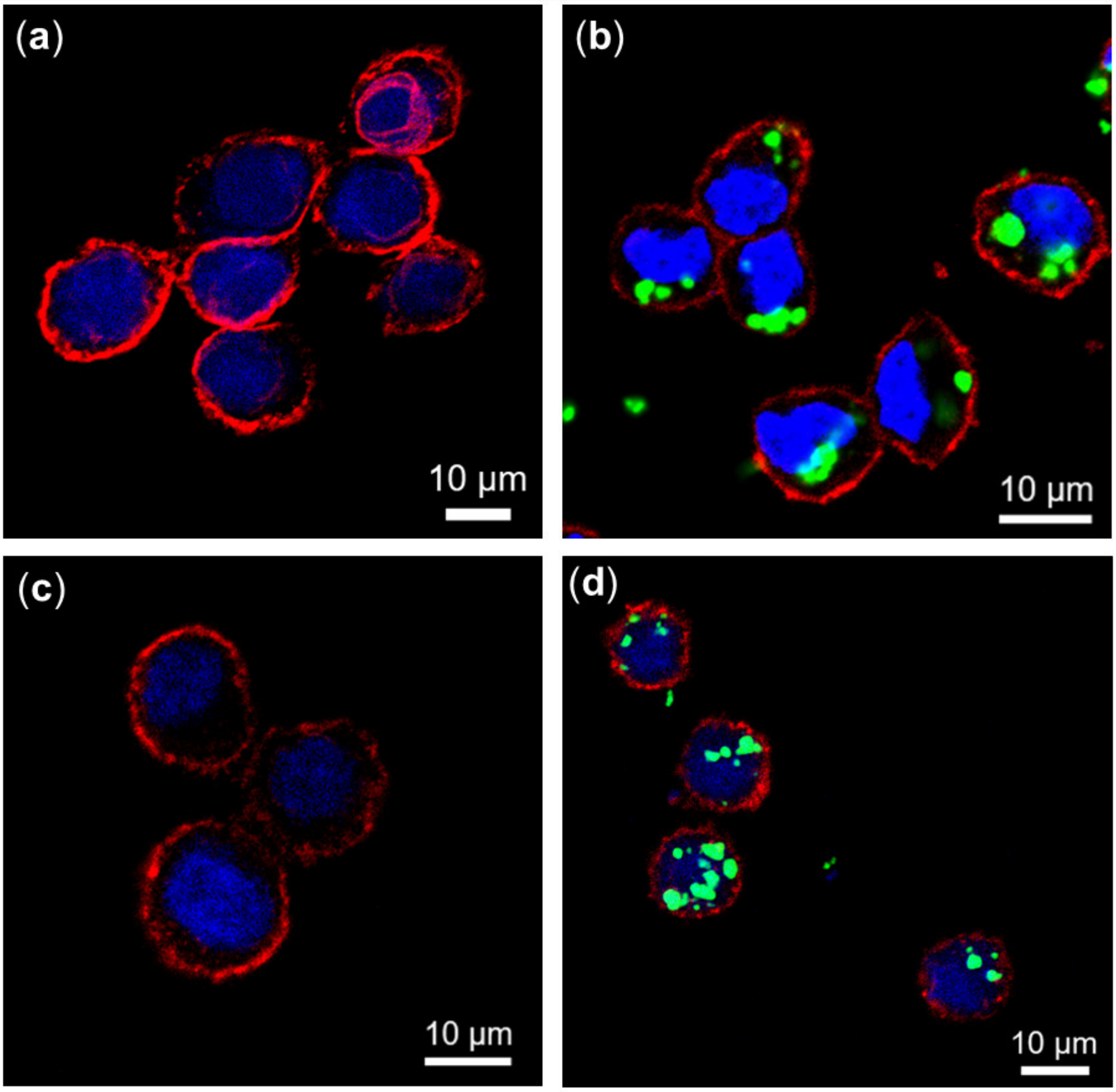

Figure S5. Confocal microscopy images of cells incubated with liposomes for 48 h at 37 °C. Top row shows GBM-CCC-002 cells incubated with (**a**) non-targeted liposomes and (**b**) PR_b-functionalized liposomes. Bottom row shows GBM-CCC-003 cells incubated with (**c**) non-targeted liposomes and (**d**) PR_b-functionalized liposomes. Liposomes with encapsulated calcein are shown in green, cell membranes in red and nuclei in blue.

Table S1. Characterization of miR-603/PEI complexes at different N:P ratios.

N:P ratio	Diameter (nm)[1]	PDI[2]	Zeta Potential (mV)
2	185.8 ± 73.9	0.56 ± 0.18	−18.9 ± 2.0
4	103.1 ± 30.2	0.43 ± 0.49	9.0 ± 1.8
6	103.9 ± 24.4	0.11 ± 0.04	20.2 ± 1.1
8	99.0 ± 16.6	0.26 ± 0.04	18.9 ± 1.6

[1] Hydrodynamic diameters are reported based on intensity from DLS. [2] Polydispersity index. Data are reported as mean ± SD; *n* =3.

Table S2. Statistical analysis comparing relative miR-603 mRNA level in GBM-CCC-001 cells following indicated treatments.

	PR_b Liposomes	PR_b Liposomes w/ miR-603	PR_b Liposomes w/ miR-603/PEI
**PBS Control**	$p > 0.05$	$p < 0.05$	$p < 0.00001$
**PR_b Liposomes**		$p < 0.05$	$p < 0.00001$
**PR_b Liposomes w/ miR-603**			$p < 0.0001$

p-values from one-way ANOVA with post-hoc Tukey's HSD test analysis comparing data shown in Figure 3a ($n = 3$).

Table S3. Statistical analysis comparing relative IGF1 mRNA level in GBM-CCC-001 cells following indicated treatments.

	PR_b Liposomes	PR_b Liposomes w/ miR-603	PR_b Liposomes w/ miR-603/PEI
**PBS Control**	$p > 0.05$	$p > 0.05$	$p < 0.0005$
**PR_b Liposomes**		$p > 0.05$	$p < 0.0005$
**PR_b Liposomes w/ miR-603**			$p < 0.001$

p-values from one-way ANOVA with post-hoc Tukey's HSD test analysis comparing data shown in Figure 3b ($n = 3$).

Table S4. Statistical analysis comparing relative IGF1R mRNA level in GBM-CCC-001 cells following indicated treatments.

	PR_b Liposomes	PR_b Liposomes w/ miR-603	PR_b Liposomes w/ miR-603/PEI
**PBS Control**	$p > 0.05$	$p > 0.05$	$p < 0.0001$
**PR_b Liposomes**		$p > 0.05$	$p < 0.00005$
**PR_b Liposomes w/ miR-603**			$p < 0.005$

p-values from one-way ANOVA with post-hoc Tukey's HSD test analysis comparing data shown in Figure 3b ($n = 3$).

Table S5. Statistical analysis comparing differences in stem cell frequency after 24 h incubation with liposomal formulations.

	PBS Control + IR	PR_b Liposomes	PR_b Liposomes + IR	PR_b Liposomes w/ miR-603	PR_b Liposomes w/ miR-603 + IR	PR_b Liposomes w/ miR-603/PEI	PR_b Liposomes w/ miR-603/PEI + IR
**PBS Control**	$p < 0.00001$	$p > 0.05$	$p < 0.00001$	$p < 0.005$	$p < 0.00001$	$p < 0.00001$	$p < 0.00001$
**PBS Control + IR**		$p < 0.00001$	$p > 0.05$	$p < 0.00001$	$p > 0.05$	$p > 0.05$	$p < 0.00001$
**PR_b Liposomes**			$p < 0.00001$	$p < 0.01$	$p < 0.00001$	$p < 0.00001$	$p < 0.00001$
**PR_b Liposomes + IR**				$p < 0.00001$	$p > 0.05$	$p > 0.05$	$p < 0.00001$
**PR_b Liposomes w/ miR-603**					$p < 0.00001$	$p < 0.00001$	$p < 0.00001$
**PR_b Liposomes w/ miR-603 + IR**						$p > 0.05$	$p < 0.00001$
**PR_b Liposomes w/ miR-603/PEI**							$p < 0.00001$

p-values as calculated using the ELDA software comparing data shown in Figure 4b (total of 24 replicate wells per cell dilution in each treatment group).

Table S6. Statistical analysis comparing differences in stem cell frequency after 48 h incubation with liposomal formulations.

	PBS Control + IR	PR_b Liposomes	PR_b Liposomes + IR	PR_b Liposomes w/ miR-603	PR_b Liposomes w/ miR-603 + IR	PR_b Liposomes w/ miR-603/PEI	PR_b Liposomes w/ miR-603/PEI + IR
**PBS Control**	$p < 0.00001$	$p > 0.05$	$p < 0.00001$	$p < 0.05$	$p < 0.00001$	$p < 0.00001$	$p < 0.00001$
**PBS Control + IR**		$p < 0.00001$	$p > 0.05$	$p < 0.00001$	$p > 0.05$	$p > 0.05$	$p < 0.00001$
**PR_b Liposomes**			$p < 0.00001$	$p > 0.05$	$p < 0.00001$	$p < 0.00001$	$p < 0.00001$
**PR_b Liposomes + IR**				$p < 0.00001$	$p > 0.05$	$p > 0.05$	$p < 0.00001$
**PR_b Liposomes w/ miR-603**					$p < 0.00001$	$p < 0.00001$	$p < 0.00001$
**PR_b Liposomes w/ miR-603 + IR**						$p > 0.05$	$p < 0.0005$
**PR_b Liposomes w/ miR-603/PEI**							$p < 0.00001$

p-values as calculated using the ELDA software comparing data shown in Figure 4c (total of 16 replicate wells per cell dilution in each treatment group).
